# COVID-19 update: the first 6 months of the pandemic

**DOI:** 10.1186/s40246-020-00298-w

**Published:** 2020-12-23

**Authors:** Giuseppe Novelli, Michela Biancolella, Ruty Mehrian-Shai, Caroline Erickson, Krystal J. Godri Pollitt, Vasilis Vasiliou, Jessica Watt, Juergen K. V. Reichardt

**Affiliations:** 1 Department of Biomedicine and Prevention, “Tor Vergata” University of Rome, 00133 Rome, Italy; 2 IRCCS Neuromed, Pozzilli, IS Italy; 3 Department of Pharmacology, School of Medicine, University of Nevada, Reno, NV 89557 USA; 4 Department of Biology, Tor Vergata University of Rome, 00133 Rome, Italy; 5 Pediatric Hemato-Oncology, Sheba Medical Center, Tel Hashomer, Israel; 6 Yale College, New Haven, CT 06520-8241 USA; 7 Department of Environmental Health Sciences, Yale School of Public Health, New Haven, CT 06510 USA; 8 College of Public Health, Medical and Veterinary Sciences, James Cook University, Smithfield, QLD Australia; 9 Australian Institute of Tropical Health and Medicine, James Cook University, Smithfield, QLD 4878 Australia

**Keywords:** Coronavirus, Pandemic, Genomics, Virus, Politics

## Abstract

The COVID-19 pandemic is sweeping the world and will feature prominently in all our lives for months and most likely for years to come. We review here the current state 6 months into the declared pandemic. Specifically, we examine the role of the pathogen, the host and the environment along with the possible role of diabetes. We also firmly believe that the pandemic has shown an extraordinary light on national and international politicians whom we should hold to account as performance has been uneven. We also call explicitly on competent leadership of international organizations, specifically the WHO, UN and EU, informed by science. Finally, we also condense successful strategies for dealing with the current COVID-19 pandemic in democratic countries into a developing pandemic playbook and chart a way forward into the future. This is useful in the current COVID-19 pandemic and, we hope, in a very distant future again when another pandemic might arise.

## Introduction

COVID-19 is an infectious disease caused by the severe acute respiratory syndrome coronavirus 2 [1]. It is currently sweeping the world almost a year on. Furthermore, it will be an enduring feature of our lives.

The COVID-19 pandemic was declared by the WHO on 11/3/2020 [2], although some countries acted sooner, for example, Australia on 24/2/2020 [3]. Therefore, we call explicitly for the examination the actions of politicians in selected countries and international organizations.

We here take stock of the current situation 6 months into the pandemic and attempt to consider the future, including relevant questions to be answered. Routes of SARS-CoV-2 transmission has been an active area of research. We discuss the prevalent modes, including the emergence of airborne transmission. Furthermore, we highlight the contribution of asymptomatic transmission of SARS-CoV-2 as a major challenge in controlling the spread of COVID-19 [4, 5].

## Main text

### The pathogen, host and the route of virus transmission

“Knowledge is a weapon. Gear up well before going into battle”, says Game of Thrones writer, George R.R. Martin. A pandemic infection such as that from SARS-CoV-2 must be fought with the weapons of knowledge: only now, after 6 months from the epidemic that has become a pandemic, do we have knowledge that can be useful to win not the battle, but the war against the SARS-CoV-2 coronavirus.

We note here that a most helpful timeline of research into COVID-19 was recently published [6]. We refer our readers to this paper rather than recapitulating the timeline here.

We will be organizing our thoughts around a series of articles to provide research advances on three important aspects of the pandemic:

1. The pathogen
2. The host
3. The routes of virus transmission

#### The pathogen

The origin and direct ancestral viruses of SARS-CoV-2 have not been identified, but it is probably a novel recombinant virus [7]. Its genome is indeed closest overall (96.2% similarity) to SARSr-Ra-BatCoV-RaTG13 from horseshoe bats (*Rhinolophus affinis*) that had been collected in Pu'er, Yunnan, China, in 2013 [8]; however, its receptor-binding domain (RBD) is closest to that of from smuggled pangolins in Guangzhou (pangolin-SARSr-CoV/MP789/Guangdong/2019). However, there might be one or more unidentified (probably bat) virus(es) with a nearly identical RBD to that of SARS-CoV-2 and pangolin SARSr-CoV. There are eight species of pangolins in the mammalian order of *Pholidota* which is most closely related to Carnivora (cat-like and dog-like carnivorans). All are insectivorous and toothless animals whose body is largely covered by keratinous scales. The human receptor for SARS-CoV-2, angiotensin I-converting enzyme 2 (ACE2), is conserved in pangolins, and coronaviruses isolated from pangolins have a receptor-binding domain in their spike protein that is uniquely similar to that of SARS-CoV-2. Interestingly, pangolins have lost *IFIH1* (interferon induced with helicase C domain 1) and *ZBP1* (Z-DNA binding protein 1) genes during evolutionary divergence. IFIH1 and ZBP1 proteins are intracellular sensors of exogeneous RNAs able to activate cellular and organismal responses, such as necroptotic cell death, interferon signaling and inflammation [9]. It is possible that the loss of the *IFIH1 and ZBP1* genes provided an evolutionary advantage by reducing the inflammation-induced damage to the host tissues and thus contributed to the transition from resistance to tolerance of viral infections in pangolins [9]. This hypothesis, if confirmed, could be the basis of the phenomenon of viral accommodation in some species. However, although there are indications of the role that the pangolin could play in the spread of SARS-CoV-2, there is no direct evidence (experimental infection) that this animal is the intermediate host of the virus [10].

The virus was fairly stable (without significant mutation) until 01/12/2020, and spread through human contact, and not from its inherent abilities for rapid growth and continuous evolution. Although 230,000 viral genomic sequences of hCoV-19 have been submitted and shared in GISAID [11], few mutations of any clinical significance have been identified thus far. A missense mutation, D614G, in the spike protein of SARS-CoV-2, predominant in Europe (954 of 1449 (66%) sequences) which is now spreading worldwide (1237 of 2795 (44%) sequences), seems to cause a higher infection rates in vitro [12, 13]. However, the lack of detailed clinical data suggests that probably this mutation is neutral. Recently, a novel recurrent mutation has been identified in Singapore and other countries, ∆382 variant, that seems to be associated with a milder infection [14]. Many other mutations have been identified and described, but none of these to date are associated with increased viral transmission. It is possible that the recurrent mutations identified are induced by host immunity through RNA modification mechanisms and probably tend to be selectively neutral, with no or negligible effects on the transmissibility of the virus [15]. Paradoxically, it could be argued that SARS-CoV-2’s low genetic diversity results in an advantage for mass vaccine immunization [16].

#### The host

The vast majority of infections are asymptomatic or mildly symptomatic, while deaths account for < 1% of infected cases. We know for certain that age is a risk factor—the death rate from COVID-19 soars for patients over the age of 60. This is true of many infections and generally is related to a deficiency in the immune system and pre-existing health conditions. Children are less prone to develop severe COVID-19 [17]. These differences are certainly attributable to the genetic diversity of the host. The scientific literature begins to give us information on these differences concerning the individual and gender production of hormones, differential T cell response (women produce more T cells compared to males), the expression of specific genes that increase or decrease their activity when the virus enters and leaves our cells, the diversity of blood groups (group A seems to increase the risk, while group 0 subjects have a lower risk of getting sick), the genes that produce our antibodies and all the defense mechanisms, as well as some genes of susceptibility to cardiovascular disease, seem to favor the severity of the disease [18, 19]. Similarly, significant association were found for HLA alleles (HLA B*27:07, DRB1*15:01, DQB1*06:02) [20]. Numerous candidate genes have now been analyzed for searching susceptibility and/or resistance alleles. Much attention has been directed to the genes that encode SARS-CoV-2 cellular entry proteins. Indeed, coronavirus entry into host cells is an important determinant of viral infectivity and pathogenesis and is an important target for host immune surveillance and therapeutic strategies [21–23]. However, none of these genes showed susceptibility and/or resistance alleles. Much attention has been directed towards the ACE2 receptor which has proved to be minimally variable and probably intolerable towards mutations with loss of function. It is possible, considering the role of ACE2 in the pathogenesis of the disease, that differences are to be found in the genes that regulate the expression of ACE2 [24]. In fact, ACE2 is expressed in many organ tissues involving vital and critical systems such as the respiratory, immunological, vascular, renal-excretory, cardiac, reproductive and nervous systems [25]. The omnipresence of ACE2 demonstrates that SARS-CoV-2 has developed an extraordinary evolutionary strategy to guarantee its replication, survival and diffusion by exploiting a very common transmembrane protein, abundant and capable of altering physiological processes in a simultaneous way.

Our genes can influence how our immune systems respond to an infection, which could explain why some people have more severe symptoms of the disease and others are asymptomatic. It is possible that a sort of “viral accommodation”, a phenomenon well known in plants and animals, exists also for coronaviruses [26]. Viral accommodation (or disease tolerance) is the ability of an individual, due to a genetic predisposition or certain behavioral (lifestyle) aspects, to thrive despite being infected with a pathogen that causes disease in a population. This is different from resistance because in this case, the infection is eliminated by the immune system or by other defensive molecules. Deciphering the molecular mechanisms of viral accommodation is important for identifying asymptomatic people and for developing drug therapies for COVID-19. To date, no specific genomic biomarkers have emerged to be used to identify subjects or groups belonging to the different risk classes and above all to be used in the prognosis and monitoring of therapies. It is certain that the consequences of SARS-CoV-2 internalization are regulated by a complex and largely unknown network of host-pathogen interactions, in which viral and host genomic variability can delimit the final outcome of the infection. For instance, little is known regarding the interaction of individual SARS-CoV-2 proteins and RNA with the host interferon (IFN) and inflammatory responses, even though such interactions can determine the fate and/or efficiency of infection, transmission, and epidemic potential of the virus. Interestingly, it was recently [27] discovered that individuals carrying germinal mutations in genes active in toll-like receptor 7 (*TLR7*) among young men with severe COVID-19 confirming an important role of type I and II IFN responses in the pathogenesis of COVID-19. This was recently supported by a study of the international consortium COVID Human Genetic Effort (covidhge.com) which revealed the presence of loss-of-function (pLOF) mutations in 13 protein-coding genes of the interferon pathway. These mutations were found in 3.5% of patients with severe disease [28]. Functional deficiency of these genes during infection greatly reduces or limits the production of type I interferon molecules. This genetic study was also reinforced by a second article from the same consortium which revealed that other severe patients have autoantibodies against interferon I for a genetic mechanism not yet identified, but which highlights the role of interferon and its receptors in the pathogenesis of severe forms of COVID-19 [29].

Despite the many unknowns that remain, there has been notable progress made towards understanding how SARS-CoV-2 spreads and infects the human host. Data reveals that the hospitalizations and deaths attributable to COVID-19 follow patterns based on a variety of factors. For instance, COVID-19 mortality data in the USA indicates that about 70% of deaths occurred in patients older than 70 years of age [30]. In addition to age, there is clear evidence that pre-existing conditions affect mortality and disease severity. Among the most prominent risk factors are a medical history of hypertension, diabetes and cardiovascular disease [31]. Diabetes is one comorbidity that is especially concerning because of the increasing prevalence of diabetes worldwide [32]. Data thus far supports the troubling link between diabetes and COVID-19. In a summary report of over 72,000 patients, the Chinese CDC found an overall case fatality rate of 2.3% which increases to 7.3% in diabetic patients [33]. One study showed that 8% of hospitalized patients were diabetic, but this percentage increases to 22% of ICU patients [34]. Furthermore, a recent meta-analysis of 21 clinical studies has shown evidence that diabetes is associated with disease severity, with the percentage of critical cases in diabetes being 44.5 % [35].

Despite an understanding of the linkage between pre-existing comorbidities and clinical outcomes, there is still much debate about the ways diabetes can impact disease severity [36]. It is known that the ACE2 receptor facilitates viral entry into the cell [22]. The binding of SARS-CoV-2 to the ACE2 receptor is notable because of the expression of the receptor in the islets of the pancreas [37]. This binding may inflict permanent damage upon the beta cells within the islet which is a cause for concern because healthy beta cells act as a central component of proper insulin secretion [38]. Beta cells are crucially important and permanently altering them is directly related to disease pathogenesis of diabetes and may be a factor in the development of post SARS-CoV-2 diabetes that has been reported in some patients thus far [39]. In addition to damage through direct binding, glycemic control is another important aspect to consider when looking at COVID-19 severity in diabetic patients; it has been shown that improved glycemic control is correlated to better patient outcomes [40]. Another factor that must be considered in diabetic patients is the downstream effects of SARS-CoV-2 binding at the ACE2 receptor. The local renin angiotensin system is of upmost importance in the pancreas for controlling insulin secretion and the ACE2 receptor is directly implicated in this system [41]. Normally, the ACE2 receptor facilitates the transition of AngII to Ang [42] which is important in maintaining balance of oxidative stress within the cell, but when SARS-CoV-2 is bound, it is unclear as to what capacity this conversion occurs [42]. While the true mechanism behind diabetes and disease severity is still unclear, what is clear is the importance of this area of developing research. Furthermore, the development of diabetes after a viral infection is not unprecedented. Indeed, when looking at past human coronaviruses such as SARS, there have also been reports of the development of diabetes [43].

#### The routes of virus transmission

SARS-CoV-2 is a respiratory virus with transmission widely accepted to occur between people in close contact, primarily through droplets expelled through various respiratory activities, such as coughing, sneezing, singing, and talking [44, 45]. There has been considerable research conducted over the past 6 months exploring the contribution of other routes of transmission to the spread of this infectious disease. Early in the pandemic, SARS-CoV-2 was demonstrated to be remain viable on surfaces for extended periods, ranging from 4 h to longer than 48 h, depending on the material [46]. More recent studies have suggested that risk of fomite transmission from inanimate objects is unclear [47]. Virus can also be found in various bodily fluids, including stool, blood, semen and ocular secretions [48]. The role of contact with these fluids in risk of transmission has yet been reported. Considerable epidemiological evidence has emerged demonstrating SARS-CoV-2 can be transmitted through the air [49, 50]. Understanding prevalent transmission routes shapes the control measures that are required to prevent and control infection with SARS-CoV-2. Acknowledgement of airborne SARS-CoV-2 transmission through inhaled aerosols was initially met with resistance by the WHO [51]. Part of the challenge in gaining recognition of this transmission mode was in [1] gathering sufficient evidence of detection of SARS-CoV-2 in the air, [2] demonstration of viability of viral material from air samples, and [3] further linking exposure of viable airborne SARS-CoV-2 with infection of COVID-19. While SARS-CoV-2 was reported in air samples collected from hospital locations and some public locations early in the pandemic (April 2020), the sampling techniques by these studies were not conducive to preserving the lipid envelope of the virus [52]. Development of air sampling technologies which collect airborne material into an aqueous media has enabled the viability of SARS-CoV-2 in air samples to be demonstrated [53]. Application of improved exposure measurement technology in different environments will allow for the dynamic nature of SARS-CoV-2-laden aerosols to be explored and high-risk settings to be identified.

Inhalation of SARS-CoV-2-laden aerosols is increasingly being recognized as a relevant transmission mode. The role of aerosols in the spread of COVID-19 is supported by [1] the range of respiratory activities, from breathing to speaking to singing, that produce aerosol and [2] the large number of cases that involve asymptomatic or pre-symptomatic individuals. Questions remain regarding the mechanisms by which aerosols are produced that lead to variability between individuals (i.e. airway geometrics between adults and children, respiratory tract fluid viscosities, stage/location of infection).

Epidemiological studies reporting COVID-19 outbreaks have commonly occurred in indoor environments (Nishiura H, Oshitani H, Kobayashi T, Saito T, Sunagawa T, Matsui T, et al: Closed environments facilitate secondary transmission of coronavirus disease 2019 (COVID-19), unpublished). As communities look to safely reopening, we can consider the characteristics of these enclosed spaces as well as the behaviors of individuals occupying these spaces to minimize the risks associated with airborne transmission. Control of expelled aerosol at the source (i.e. mouth/nose of infected individuals) by wearing face coverings (masks) is an important strategy for reducing transmission. Virus-laden aerosol can remain suspended in the air for hours and be easily transported some distance away from infected individuals. Aerosol concentrations in indoor spaces can be effectively lowered through properly ventilation (i.e. use of outdoor air, high air changes) and filtration of recirculated air. Ultimately, these strategies aimed at airborne transmission should be layered together with the fundamental principles of disease control (i.e. physical distancing, hand hygiene) when developing a safe and comprehensive plan for limiting community transmission of COVID-19.

### Therapies

Since the pandemic broke out, international research laboratories, together with pharmaceutical companies and biotech companies, are working in an unprecedented manner at extraordinary rates to find and evaluate drugs, vaccines and other solutions aimed at decreasing hospital admissions and to help heal patients and support recovery. Regulatory agencies worldwide (EMA, FDA, PMDA) have been increasingly enriched with new trials against the disease caused by the SARS-CoV-2 virus. Antiviral drugs are being studied, aimed at inhibiting the replication of the virus; immunomodulatory drugs, to attenuate the overactive immune system; neutralizing antibodies, to inhibit the virus and help the immune system clear the infection; and passive immunization with convalescent serum which contains a wide range of antibodies, cytokines and other immunomodulators. Dozens of “repurposed” drug candidates (most notably, antiretroviral protease inhibitors approved for the treatment of HIV) are still being evaluated in hundreds of trials around the world [54]. Many have been eliminated because they are ineffective. Remdesivir, on the other hand, currently seems to be the only treatment at the moment with some effectiveness against COVID-19 (albeit with not striking results) [55]. Recently, British colleagues have shown that dexamethasone, a steroid anti-inflammatory, is effective in severely infected patients who develop a systemic inflammatory response resulting in lung injury and dysfunction of other organs [56]. However, this drug cannot be used for all patients, who may indeed have very serious adverse events. As for the immunomodulator, tocilizumab, the results are mixed and it is necessary to await further evaluations. There is much discussion on the possibility of using the plasma of convalescents, which is the plasma that is collected from subjects exposed to the virus and who have developed antibodies following the resolution of the infection. Passive administration of these antibodies via convalescent plasma transfusion may be a short-term strategy to confer immediate immunity to sensitive individuals. Convalescent plasma has been used successfully in the past as post-exposure prophylaxis and in the therapeutic treatment of other coronavirus outbreaks (e.g. SARS-1 and MERS-CoV]) and other autoimmune and chronic inflammatory diseases, such as dermatomyositis, Kawasaki disease, multiple sclerosis, lupus, chronic lymphocytic leukemia, and idiopathic thrombocytopenic purpura. Convalescent plasma has also been used in the COVID-19 pandemic in a few published cases in China, Italy and other countries with some clinical benefits documented at radiological, laboratory level and improved survival [34]. Nevertheless, these experiences suggest that we need further studies characterized by a standardization of quality and quantity of antibodies. In fact, [57] demonstrated that (a) individuals with mild and severe disease produced neutralizing IgG to SARS-CoV-2 10 days after disease onset, (b) SARS-CoV-2 persisted longer in those with severe disease, and (c) there was cross-reactivity between antibodies to SARS-CoV-1 and SARS-CoV-2, but only antibodies from patients with COVID-19 neutralized SARS-CoV-2. Not very different is the idea of using, for passive immunization, monoclonal antibodies (Mabs) obtained in the laboratory instead of the plasma of convalescent subjects [58]. Monoclonal antibodies are specifically addressed to block the spike protein of the virus in order to block its binding with the ACE2. Mabs are preferred because of their specificity, purity, low risk of blood pathogen contamination, and safety over convalescent plasma therapy. Passive administration of monoclonal antibodies could have a major impact on the control of the SARS-CoV-2 pandemic by providing immediate protection, complementing the development of prophylactic vaccines. There are at least 15 groups in the world active in the identification and production of monoclonal antibodies against SARS-CoV-2. The passive infusion of Mabs can be used as pre-exposure or post-exposure prophylaxis and offer immediate protection against infection that could last weeks or months. Even if a vaccine will be available, the weeks to generate an effective immune response emphasizes the benefits of passive immunity from Mabs in a variety of circumstances including, for example, healthcare settings such as hospitals and nursing homes, or health centers, high risk industries or cruise ships.

Currently, there is no single specific vaccine or effective antiviral therapy against SARS-CoV-2. Companies at work estimate that this vaccine will take years to develop and test before reaching a large population [59]. There are currently over 200 different candidate vaccines being studied, none of them have yet proved effective and safe. Recently, at least 10 companies announced the first data from their phase 3 studies of vaccine candidates with promising and interesting results. Some of these companies have begun the data evaluation process at the Food and Drug Administration (USA) and European Medicine Agency (EMA) [60]. However, it is important that pharmaceutical companies who have announced data on their vaccines in press releases provide the scientific community with the results as soon as possible through publication in peer-reviewed journals. The world looks forward to this data, and we will review progress on this front in due course when full peer-reviewed data becomes available. We all deserve transparency in our search for safe and effective vaccines. Interestingly, two novel candidate vaccines based on mRNA which encode a secreted SARS-CoV-2 receptor-binding domain encapsulated in lipid nanoparticles have been obtained [61]. The manufacturers of these vaccines (Moderna and Pfizer) have announced that their vaccines would have between 90 and 95% efficacy. But it is necessary to proceed with caution at least until phase 3 is concluded. In addition, it will be non-trivial to reach some populations since these vaccines are RNA-based and hence need low temperature storage.

On 11 and 18 December, respectively, the FDA authorized the administration of the Pfizer / BioNTech and Moderna vaccines.

Having no vaccines or targeted therapies yet, effective solutions are quickly needed, also in consideration of the economic and social recovery phase, and to allow hospitals to operate at full capacity. To win this war on a global level, the answer must be global: the responsibility of the individual in adopting precautionary behaviors must be combined with consistent indications, cooperation between institutions, circulation of knowledge and strong signs of support for research because the answer can only be found by searching for it thoroughly.

### A pandemic playbook

The COVID-19 pandemic has led to various strategies to prevent and contain its spread. We condense here strategies from Western democracies into a playbook which may be useful now and in the future. Western democracies have expectations of civil liberties that must be weighed against the common good in an emergency situation.

We take particular note of Mike Ryan’s (of the WHO) suggestion to “go hard and go early” [62] which appears to be particularly useful. Furthermore, we must learn to live with the COVID-19 pandemic for now.

We propose here a pandemic playbook for both early and less severe stages designed to limit community transmission in earliest stages to control COVID-19 and future pandemics (Table 1), as well as more drastic action required for more severe stages (Table 2). Ultimately, in either scenario, the virus must be controlled. Public health still has a useful tool to manage and organize response strategies to pandemics: the reproduction number, R0, which provides the average number of new cases of a disease arising from a single case. Its simple mathematical power is still relevant today [63].

**Table 1:** The Pandemic Playbook I: leaders must act decisively and early, informed by science

The Pandemic Playbook I: leaders must act decisively and early, informed by science	
Close borders as quickly as possible	
Isolate incoming travellers in supervised quarantine	
Require physical distancing	
Encourage good hygiene, e.g. frequent hand washing, use of alcohol hand sanitizers as an alternative, cough etiquette like coughing and sneezing into a disposable tissue etc., as warranted	
Test quickly, rigorously and widely	
Deliver test results as quickly as possible, ideally in 24–48 h, to advise affected individuals, contacts etc. in a timely fashion	
Contact trace every case and require close contacts to self-isolate (or quarantine if advisable)	
Take care of the most vulnerable, e.g. in aged care by restricting access	
Limit gatherings, to 10 or the like, as the situation dictates	
Consider limiting transportation, domestic borders etc.	
Communicate clearly, age-appropriately and addressing ethnic and other minority groups appropriately	
Prepare (and possibly expand) hospital facilities, incl. beds, ICUs, staffing for a possible onslaught of severely affected patients	
Be prepared for the unexpected

**Table 2:** The Pandemic Playbook II: during a (raging) pandemic

The Pandemic Playbook II: During a (raging) pandemic	
Leaders must continue to act decisively and early, informed by science	
Test quickly, rigorously and widely	
Maintain quick turn-around on test results	
Contact trace as effectively as possible, incl. using of technology (such as a tracing app)	
Close contacts must self-isolate if not quarantine	
Enforce self-isolation, e.g. through door knocking repeatedly, and consider supervised quarantine if need be	
Restrict movement, e.g. to 5 km in cities, 20 km in suburbia, 50 km in the country	
Rigorously ring fence hot spots, if feasible, incl. local and other domestic borders	
Transport, except for essential business, must be limited	
Require social distancing (Table 1)	
Limit gatherings, to 2 or the like as the situation dictates	
Allow only essential business to stay open, incl. supermarkets, pharmacies, doctors and the like	
Limit trips outside the home for food, healthcare or the like each day within a prescribed radius and to 1x/d	
Require protective equipment as warranted, e.g. appropriate face coverings	
Advise of the need for proper hygiene (Table 1)	
Communicate clearly, age-appropriate and addressing ethnic groups appropriately	
Ensure the best possible care for the most vulnerable	
Manage hospital capacity wisely, incl. staff (which may fall sick), beds, ICU etc.	
Be on the lookout for the unexpected and unexplained	
In more dire circumstances, consider also:	Curfews
	Isolation of cases and perhaps contacts etc. (e.g. on islands)

Reopening societies, economies and countries safely after the COVID-19 and other pandemics is also a great challenge. It must avoid additional waves while allowing a return to normality perhaps with some restrictions. Clearly there are great physical, mental and economic benefits. We believe that definitive statements on successes cannot yet be made at this point in time, especially for democracies. However, the situation in Europe was recently reviewed [64]. In any case, we attempt to collate some suggestions in Table 3 for this and future pandemics. The virus must be contained, suppressed if not eliminated at this stage.

**Table 3:** The Pandemic Playbook III: reopening

The Pandemic Playbook III: *Reopening*	
Reopen cautiously with a measured and careful approach	
Continue testing rigorously and widely	
Ringfence as soon as necessary if localized outbreaks are documented	
Be prepared for the unexpected	
Vaccinate widely if possible	

## Conclusions: outlook in context, including the political landscape

The COVID-19 pandemic will hopefully pass in a couple of years [65]. In the short term, we should learn to live safely with the disease and specifically do what is necessary to minimize harm due to COVID-19 each one of us and as a society. Next, in the medium term, we will hopefully have a vaccine which must be accessible to all people of this planet. Additionally, we may develop improved treatments for COVID patients which would be another weapon in fighting the pandemic. We should also learn the necessary epidemiologic, medical, scientific and political lessons. Clearly, we can also not neglect other health conditions that require medical attention [66].

Beyond that, we should hold our politicians to account. Scientific research and medical practice will contribute throughout with essential information and hopefully treatments and vaccines along with novel biological, epidemiologic, medical and other insights. As noted and in due time, we must hold politicians accountable for their actions. In Western democracies, we can and should do so at the ballot box. Furthermore, we should also hold international organizations, and their nowadays mostly political leaders, funded by us taxpayers, to account to us, the taxpayers funding their actions. Holding national and local public officials is our privilege as citizens we must exercise it in these challenging times. Advising, when called upon, is our duty as citizen-scientists.

Functioning international organizations with expert leadership taking in scientific advice are key. Unfortunately, many of these organizations are influenced by geopolitical interests that often delay or prevent rapid and effective intervention as is required in a pandemic. The sudden appearance of a new pathogen has highlighted some critical issues, weak links in the structure of our society that have contributed to making us vulnerable to the pandemic. In fact, during the COVID-19 emergency, the problem of determining an orderly and clear structure of the competences of the bodies was proposed, with heavy implications on the management system of health plans in the various countries and on that of a more purely legal nature—central, regional or decentralized administration—deputies to safeguard and guarantee the fundamental right to health. This was also evident initially in Wuhan, Hubei province, where the pandemic originated, in central mainland China. Delays, accountability and improvisation have unfortunately favored an emerging epidemic becoming the first pandemic of the twenty-first century.

It is clear that from this experience that we must all commit ourselves to reaping benefits for the future, avoiding the repetition of mistakes and relying on technical and scientific skills. In fact, it is appropriate that the whole system of health institutions can outline strategies aimed at a long-term control of COVID-19, strategies based on innovative methods useful for estimating and stratifying the susceptibility to the risk of infections. The shared purpose, the one to which every subject called in these months to offer their contribution aims, is the prevention of the impact of pandemics—the current one and others possibly the future ones—and more immediately to trust in science and medical practice to have a short drugs and vaccines capable of neutralizing SARS-CoV-2, responsible for COVID-19.

Finally, we plan to prepare timely updates on the COVID-19 pandemic for publication in this journal probably with the next 1 year into the current COVID-19 pandemic.

## Data Availability

Data sharing is not applicable to this article as no datasets were generated or analysed during the current study.

## Abbreviations

COVID-19: Coronavirus disease 2019
EU: European Union
FAO: Food and Agriculture Organization
ICU: Intensive Care Unit
IFN: Interferon
Mabs: Monoclonal antibodies
SARS-CoV-2: Severe acute respiratory syndrome coronavirus 2
UN: United Nations
UNESCO: United Nations Education, Scientific and Cultural Organization
WHO: World Health Organization
EMA: European Medicines Agency
FDA: Food and Drug Administration
PMDA: Pharmaceuticals and Medical Devices Agency
